# The Role of Prophylactic Central Neck Dissection in Differentiated Thyroid Carcinoma: Issues and Controversies

**DOI:** 10.1155/2011/127929

**Published:** 2011-09-29

**Authors:** Kai-Pun Wong, Brian Hung-Hin Lang

**Affiliations:** Division of Endocrine Surgery, Department of Surgery, Queen Mary Hospital, The University of Hong Kong, Pokfulam Road, Hong Kong

## Abstract

Prophylactic central neck dissection (pCND) in differentiated thyroid carcinoma (DTC) is one of the most controversial surgical subjects in recent times. To date, there is little evidence to support the practice of pCND in patients with DTC undergoing total thyroidectomy. Although the recently revised American Thyroid Association (ATA) guideline has clarified many inconsistencies regarding pCND and has recommended pCND in “high-risk” patients, many issues and controversies surrounding the subject of pCND in DTC remain. The recent literature has revealed an insignificant trend toward lower recurrence rate in patients with DTC who undergo total thyroidectomy and pCND than those who undergo total thyroidectomy alone. However, this was subjected to biases, and there are concerns whether pCND should be performed by all surgeons who manage DTC because of increased surgical morbodity. Performing a unilateral pCND may be better than a bilateral pCND given its lower surgical morbidity. Further studies in this controversial subject are much needed.

## 1. Introduction

Differentiated thyroid carcinoma (DTC) accounts for over 90% of all follicular cell-derived thyroid malignancies and is the commonest primary endocrine-related malignancy. In our locality, its age-adjusted incidence has doubled over the last 25 years, and a similar trend has been observed elsewhere [1]. Despite this, the cancer-specific mortality remains low with an overall 10-year survival above 90% [2]. However, recurrent/persistent disease after seemingly curative surgery remains a major cause of patient morbidity and poses a management challenge for clinicians [3, 4]. Furthermore, clinicians have witnessed a paradigm shift in the definition of cure from not dying of the disease to being “completely disease-free” with good quality of life and to, more recently, having undetectable postsurgical stimulated thyroglobulin (Tg) levels [5–8]. However, despite our best effort, overall 15 to 30% of patients with DTC develop locoregional recurrences after successful initial operation leading to increased patient morbidity. Numerous studies have shown that these recurrences most commonly involve the locoregional lymph nodes [3, 4, 9]. In terms of lymph node metastases, at the time of first presentation, up to 20–30% patients with PTC would have gross involvement and approximately 90% would have microscopic involvement (i.e., micrometastases) [7, 10]. Therefore, lymph node metastases in PTC is very common, but it is only over the last 10–15 years that lymph node metastasis is recognized to be associated with a poorer cancer-specific survival [7, 11–15]. With better understanding of the lymphatic drainage of the thyroid gland, it is now believed that lymphatic metastasis of DTC tends to occur in the ipsilateral central compartment as the first place for metastasis. From there, it next spreads either to the ipsilateral lateral compartment or contralateral central compartment [16–18]. In attempt to further improve the management of lymph node metastases, the role of prophylactic central neck dissection (pCND) has been extensively studied over the past 5–10 years. For surgeons, pCND has become one of the major research focuses. In year 2006, American Thyroid Association (ATA) published a guideline on management of thyroid cancer and suggested that “pCND should be considered for patients with papillary thyroid cancer” [19]. This recommendation was based on the association between lymph node metastasis, recurrence, and survival. It was also based on the assumption that pCND would reduce the risk of recurrence and perhaps improve cancer-specific survival. However, this recommendation was criticized as being vague and imprecise, and it also generated much controversy among the surgical community. In the revised guideline by ATA in 2009, they recommended a much more risk-orientated approach and suggested that “pCND may be performed, especially in patients with advanced primary tumors” and “total thyroidectomy without prophylactic central neck dissection may be appropriated for small (T1 or T2), non-invasive, clinically node negative patients [7].” With changes in attitude on pCND over recent years, the authors believe it is timely to examine the role of pCND at the time of thyroidectomy in patients with DTC. In the present review, the authors would focus on the potential benefits and morbidities of pCND and examine some of the arguments for and against pCND and its implication on future treatment.

## 2. Definition of a Central Neck Dissection (CND)

Despite numerous reports describing the technique of performing a CND, there were inconsistencies regarding the definition of what is a CND and what constitute the central neck compartment [17, 18, 20, 21]. An example of this inconsistency would be the definition of the upper extent of CND. Some surgeons would describe it as inferior thyroidal artery [20, 22], while others would describe it as superior thyroid artery [23] or more commonly hyoid bone [24–28]. Furthermore, as a result of these inconsistent descriptions and terminologies on CND, at times, it is difficult to assess the results on CND in different studies. With the contribution of experts from endocrinology, endocrine surgery, otolaryngology, head and neck surgery, and radiology, the working group within the ATA published a guideline focusing on relevant anatomy and nodal subgroups of central compartment and defining a consistent terminology to facilitate consistent reporting on operation records and publications [29].

**Figure 1:**
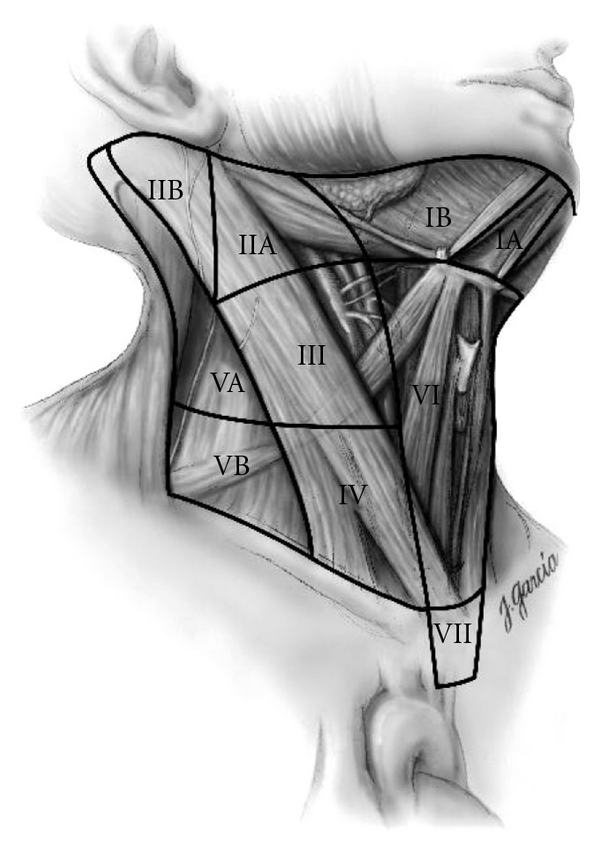
Schematic right anterior oblique view indication levels of the neck and upper mediastinum relevant to neck dissection [29] (reproduced with permission).

In the recent ATA guideline, the central neck compartment is described as bounded by hyoid bone (superiorly), carotid arteries (laterally), superficial layer of deep cervical fascia (anteriorly), and deep layer of deep cervical fascia (posteriorly). It is also referred as level VI if inferior boundary was defined as sternal notch, although, in the literature, the term “central neck compartment” and “level VI” have been used interchangeably [30, 31]. In thyroid gland, lymphatic vessels communicate with anterior superior mediastinum, which can be accessible by cervical approach, ultimately to thoracic duct. To achieve adequate lymphatic excision, inferior border of central compartment is defined by the innominate artery on the right and corresponding level on left. However, some would refer this area as level VII [30] (Figure 1). In terms of nodal groups, they include prelaryngeal (Delphian), pretracheal, and at least one para-tracheal lymph nodal basin. Either unilateral or bilateral paratracheal central neck dissection could be performed and designated in the operation record. Comprehensive compartmental neck dissection does not include “berry-pricking” or limited neck dissection as they are usually associated with higher rate of recurrence [32, 33].

ATA working group also developed a consensus on terminology on intention of central neck dissection. A CND can either be therapeutic (i.e., nodal metastasis detected clinically or radiologically before or during operation) or prophylactic (nodal metastasis was not detected clinically or radiologically). This is an important distinction because few, if any, surgeons would disagree on performing a CND if it was done for a therapeutic intent whereas the major controversy remains in the prophylactic intent. According to the recent recommendation, apart from stating the intent, a description of unilateral (removal of prelaryngeal, pretracheal, and one paratracheal nodal basin) or bilateral (removal of prelaryngeal, pretracheal, and both paratracheal nodal basin) CND should be clearly documented. With standardization on description of indication and extent of neck dissection, communication between health care workers should improve and that serves as a good foundation for future clinical studies.

## 3. Arguments for pCND in DTC

Table 1 summarizes some of the arguments for pCND in DTC. One of the arguments for pCND is that central lymph node metastasis is very common and may have certain prognostic significance. As mentioned earlier, gross lymph node metastasis is present in up to 20–50% of the patients with standard pathological techniques and micrometastasis is present in 90% of lymph nodes [10, 19]. Up to 15 to 30% of patients with PTC would develop recurrence after successful thyroidectomy secondary to lymph node metastasis [3, 4, 9]. Earlier studies suggested that there was an association between presence of lymph node metastasis and local recurrence but not cancer-specific mortality [34]. Wada et al. reported a study of 134 patients and showed that patients with lymph node metastasis had a significantly higher rate of recurrence (16.3% versus 0%, *P* < 0.05). Clinically apparent lymph node in addition was more prone to have recurrence (24% versus 7.6%) [35]. This finding is supported by other studies [36]. A case series with longer follow-up period similarly reported lymph node metastasis at presentation associated with increased risk of recurrence but not cancer-specific mortality [37].Various risk stratification systems assessing survival such as MACIS [38], AGES [39], and AMES [40] have deliberately left out lymph node metastasis as a parameter. As a result, the importance of lymph node metastasis affecting survival was underemphasized in the earlier period. However, more recent large-scale studies suggested that lymph node metastasis may have a negative effect on survival. Mazzaferri and Jhiang reported a study of 1300 patients with 30 years of followup and found that lymph node metastasis was associated with increased cancer-specific mortality in 20-year time (10% versus 6%) [13]. According to Surveillance Epidemiology and End Results database comprising 19,918 patients, there was a significant but small difference in survival between patient with or without lymph node metastasis [14]. For patients with lymph node metastasis, the 14-year survival rate was 79% and 82% in nonmetastatic patients (*P* < 0.05) [14]. Another large population-based case control study with 5123 patients over a 30-year period shows 595 patients who died of thyroid cancer [15]. Predictors for cancer-related deaths included distant metastasis (odd ratio (OR) 6.6, 95% confidence interval (CI) 4.1–10.5), incomplete resection (OR 4.2, 95% CI 3.1–5.6), and lymph node metastasis (OR 1.9, 95% CI 1.1–3.6) [15]. Presence of nodal metastasis was also correlated to persistence and recurrence of PTC [11, 12].

The second argument for pCND is that central lymph node metastasis cannot be reliably diagnosed preoperatively, especially when the thyroid gland has not been removed. Although the use of ultrasound is now regarded as a standard preoperative workup modality in DTC, detection of central neck lymph node metastasis with preoperative ultrasound examination remains difficult [41]. Preoperative ultrasound in detection of lymph node metastasis has a high specificity (92%) and positive predictive value (81–92.1%), but low sensitivity (51–61%) and negative predictive value (63.4–76%) [42, 43]. And it is not sensitive to detect central compartment metastasis with ultrasound (sensitivity of 30.0% versus sensitivity of 93.8% in lateral neck metastasis) [44]. Because of the inaccuracy of preoperative ultrasound, pCND has been advocated. The reasons for this inaccuracy include, firstly, these abnormal central lymph nodes are often small in size [45] and, secondly, the location of these lymph nodes is often deep down in the neck just posterior to the sternum making the ultrasound beam difficult to reach [44]. 

The third argument is that those without pCND are at higher risk of central neck recurrence and central neck reoperation is associated with a higher morbidity than pCND when done at the time of initial total thyroidectomy. With more frequent and sensitive surveillance modalities available (e.g., stimulated Tg levels), earlier local recurrence or metastasis is now being able to be diagnosed. Despite its unknown and somewhat controversial clinical significance, reoperation over level VI lymph node and thyroid bed near tracheoesophageal groove is often advocated because of fear for further local invasion (e.g., to recurrent laryngeal nerve) and distant metastasis. But it is associated with significantly increased morbidity in the reoperative setting [46]. Secondary to scar tissue and distortion of anatomy, recurrent laryngeal nerves and parathyroid glands are generally more difficult to be identified [47, 48]. In a small series, in up to 15% of cases, the recurrent laryngeal nerve failed to be located intraoperatively and that resulted in unintentional nerve injury [49]. Segal et al. reported a retrospective review of 503 patients and showed a higher rate of permanent recurrent nerve injury (5.8% versus 25%) and permanent hypoparathyroidism (5.0% versus 8.3%) in reoperation compared with primary operation [50]. Beside recurrent laryngeal nerve, parathyroid gland was also at risk. Other studies found similar findings [18, 51–53]. Devascularization of glands and accidental removal of parathyroid gland occur commonly in central neck reoperation because it is difficult to differentiate a metastatic lymph node from a parathyroid gland and there is a tendency of more extensive dissection in recurrent cases [47, 54]. With pCND performed in the initial operation, it obviates the need or at least reduces the chance of returning to the operated field. Therefore, initial pCND may avoid the significant morbidity associated with central neck reoperation.

## 4. Argument against pCND in DTC

Table 1 lists some of the arguments against pCND in DTC. Firstly, in a true prophylactic setting, only small and subclinical metastatic lymph nodes would be identified at the time of operation [55]. One study has found that most of these involved lymph nodes were subclinical and ≤5 mm in size (66%) [56]. For most surgeons, it is difficult to believe that they would ultimately result in significant poor prognosis as macroscopic metastasis in the therapeutic setting would. Furthermore, as reported previously, microscopic metastasis is present in central compartment in up to 63% [6], but the recurrence rate remains acceptably low in this situation (1.3–6%) [3, 28]. Although there is no strict definition of defining micrometastasis, it seems that there is a real difference between presence of microscopic metastasis and macrometastasis on future recurrence. This was somewhat confirmed by a few studies, mostly from Japan. Wada et al. reported their study on 259 patients with papillary microcarcinoma of thyroid about their recurrence with 53-months (mean) followup. For patients undergoing therapeutic neck dissection, the recurrence rate was 21%. While only 0.43% patient undergoing pCND developed recurrence despite high microscopic lymph node involvement rate (61%) [57]. In addition, in a subgroup of patients without clinically or radiologically overt metastasis that has not undergone a central neck dissection, the locoregional recurrence rate was acceptably very low (0.65%) [57]. Gemsejäger et al. reported a consistent and similar finding on their study of 159 patients with papillary thyroid cancer in 2003 [58]. After mean follow up of 8.1 years, central neck recurrence rate was 7.1% and 0% in patient with therapeutic and pCND, respectively. For patients indicted but not having pCND, the recurrence rate was also 0% [58]. Furthermore, it is now believed that most of these micrometastases are expected to be controlled or ablated by radioiodine (RAI) ablation under thyroid stimulating hormone stimulation [8]. For patients harboring central neck compartment micrometastasis, pCND dissection only provides a small benefit in terms of recurrence, especially in those with papillary microcarcinoma and the overall prognosis might not change after removal of nonpalpable microscopic metastatic lymph nodes [5, 23, 57].

Some authors are concerned that pCND would be widely adopted when its efficacy has been confirmed [55]. The potential benefits of pCND should be balanced against the possible increased morbidity. Some have questioned on the applicability of pCND in the wider surgical community as not all surgeons would have same surgical experience. Low morbidity rate in pCND was reported while the operation was done by experienced surgeons [6, 8, 23]. However, more than half of the operations on patients undergoing thyroidectomy were performed by surgeons who performed less than 5 cases per year in US [59]. Like other types of surgery, the rate of complications is inversely related to the volume of surgery done in that particular center or by that particular surgeon [60, 61]. Therefore, the surgical morbidity is expected to be significantly higher in low-volume surgeons. Another point worth noting is the fact that although central neck reoperation might be associated with higher morbidity than initial pCND, Shen et al. has argued that in “experienced” hands, a central neck reoperation could also be performed as safely as a nonreoperated CND [47]. 

## 5. A Literature Review of Survival Outcomes and Morbidities of pCND in DTC

To date, there is little good evidence to show that pCND improves cancer-specific survivals in DTC.  Table 2 shows a literature summary of studies which specifically evaluated cancer-specific survival between thyroidectomy alone or thyroidectomy with pCND. To date, the strongest evidence supporting the role of pCND in DTC came from an earlier study carried out by a Swedish group. Tisell et al. [28] evaluated 175 patients who underwent thyroidectomy with CND and compared with contemporaneous controls from other two studies of Scandinavian population conducted on patients in Norway and Finland [62, 63]. They showed that patients who underwent thyroidectomy with pCND had a higher survival rate (1.6% versus 8.4–11.1%). However, these studies were criticized for their lack of no statistical comparison between two different study periods (Swedish: 1970–1989 [28]; Finland: 1956–1979; Norway: 1970–1989) and for their difference in rate of curative surgery [62–64]. 

Another important outcome parameter or survival surrogate is disease recurrence or disease-free survival.  Table 3 shows a literature summary of studies which evaluated recurrences between thyroidectomy alone or thyroidectomy with pCND. There have been great variations in terms of indication (therapeutic or prophylactic), extent (unilateral or bilateral), and duration of followup among different studies. The majority of studies did not show any significant differences. Moo et al. reported a decrease in recurrence rate in patients who underwent bilateral pCND (4.4% versus 16.7% *P* = 0.13) [24]. Zungia and Sanabria analyzed a cohort of 266 patients with 6.3 years mean followup and reported the 5-year disease-free survival was comparable (88.2% versus 85.6%; *P* = 0.72) [65]. Interestingly, a recent meta-analysis comprising 5 retrospective comparative studies (*n* = 1264) found that there was an insignificant trend toward lower overall recurrence rate in the group who underwent either unilateral or bilateral pCND when compared to those who had total thyroidectomy only (2.02% versus 3.92%, odds ratio = 1.05, 95%CI = 0.48−2.31) [66]. Their subgroup analysis revealed no decrease in central (1.86% versus 1.68%, OR 1.31, 95% CI 0.44–3.91) or lateral compartment recurrence (3.73% versus 3.79%, OR 1.21, 95% CI 0.52–2.75) [66]. Therefore, based on these findings, there might be a potential benefit of lower recurrence in those who underwent either unilateral or bilateral pCND, although larger-scale prospective studies are required to confirm this.

Serum Tg level is useful in detecting persistent or recurrent DTC after thyroidectomy and RAI ablation [9]. A detectable postsurgical Tg level is associated with risk of recurrence, and so it may be applied as an surrogate marker of outcome in studying prognosis of DTC [7, 12]. Sywak et al. examined 447 patients with clinically node-negative papillary thyroid carcinoma, while 56 patients underwent thyroidectomy plus pCND [5]. Though there was no significant difference in recurrence or survival after a short-median followup (thyroidectomy plus central neck dissection versus thyroidectomy alone: 25 versus 70 months), they showed that there was a significantly lower level of stimulated Tg at 6 months after RAI ablation (mean: 0.41 versus 9.3, *P* = 0.02) and higher proportion of athyroglobulinemia (72% versus 43%; *P* < 0.001) [5]. In contrast, Hughes et al. found that there was no difference in postablation median-stimulated Tg level or rate of athyroglobulinemia between patients undergoing thyroidectomy with or without bilateral pCND [6]. However, in their subgroup analysis of patient undergoing central neck dissection, they demonstrated that preablation Tg level was significantly higher in node-positive patient, these patients achieved a comparable rate of postablation athyroglobulinemia after a higher dose of RAI [6]. More recently, Lang et al. retrospectively analyzed 185 patients with PTC and of these, 82 (44.3%) patients had an unilateral pCND together with a total thyroidectomy (CND+ group) [8]. They found that the CND+ group had a significantly lower median preablative-stimulated Tg level (<0.5 ug/L versus 6.7 ug/L, *P* < 0.001) and achieved a higher rate of preablative athyroglobulinemia (51.2% versus 22.3%, *P* = 0.024) than those who underwent a total thyroidectomy only, but these differences were not observed 6 months after ablation. They also found that pCND was the only independent factor for preablative athyroglobulinemia [8]. In their experience, most of the residual microscopic disease, presumably not removed by the initial pCND, was still able to be ablated by RAI ablation, and so the group without pCND achieved similar stimulated Tg levels and similar rate of athyroglobulinemia 6 months after ablation [8]. The authors concluded that although performing pCND in total thyroidectomy may offer a more complete initial tumor resection than total thyroidectomy alone by minimizing any residual microscopic disease, such difference becomes less noticeable 6 months after RAI ablation [8].

Increased patient morbidity is one of the major concerns. Increased risk of transient hypoparathyroidism has been consistently shown in many studies [5, 6, 8, 22–24, 27].  Table 4 shows a comparison of patient morbidities between CND+ and CND− groups. The higher rate of temporary hypoparathyroidism could be explained by the higher rate of unintentional removal of parathyroid glands (i.e., unintentional parathyroidectomy) and subsequent autotransplantation [5, 6, 8]. Unintentional devascularization of parathyroid glands during dissection also contributes to the higher rate of temporary hypoparathyroidism. In terms of temporary recurrent laryngeal nerve injury, Palestini et al. reported a higher rate transient recurrent laryngeal nerve injury in patients undergoing thyroidectomy plus bilateral neck dissection (1.4% versus 5.4%, *P* < 0.05) [22], while other studies failed to show any statistically significant differences [5, 6, 8, 23, 25, 27]. To date, no studies have shown an increase risk of permanent hypoparathyroidism or recurrent laryngeal nerve injury. A recent systematic review comprising 5 retrospective studies evaluated the morbidity of pCND and found that there was one extra case of transient hypocalcaemia for every eight pCNDs performed [68]. However, there was no increased risk of permanent hypocalcaemia, transient or permanent recurrent nerve injury [68].

## 6. Implications on Overall DTC Treatment

Even though it is generally agreed that large-sized DTC, extrathyroidal extension, or lymph node metastasis would benefit from postsurgical radio-iodine ablation, the decision for RAI ablation in DTC less than 2 cm in diameter remains less clear and it mostly depends on the lymph node status [7]. pCND could provide more accurate pathological staging because of better known central compartment lymph node status (i.e., N0 or N1a instead of Nx) and also more frequent administration of RAI ablation because of greater incidence of N1a. From previous studies, up to 30% of DTCs were upstaged as a result of pCND [6, 8, 26]. Even in the absence of extrathyroidal extension or other prognostic factors, pCND affects the final tumor stages of patients aged over 45 years old because tumors are upstaged from stage I/II to III based on the stage groupings in the AJCC/UICC TNM staging system [71, 72]. In terms of survival, they would be classified as stage III with 10-year survival close to 85–90% while those patients younger than 45 would still remain as stage I with 10-year survival rates of more than 95% [2, 73]. This obvious stage migration is an issue which surgeons should be aware of. On the long run, this may translate into an overall improvement in cancer-specific survival, particularly tumors belonging to stages I-III, because similar risk DTCs are being upstaged [72].

For low-risk patients, RAI ablation at best only provide minimal benefits in terms of recurrence or survival [7]. Ross et al., in retrospective analysis of 611 patients with papillary microcarcinoma, found that RAI ablation did not reduce recurrence rate in patients with nodal metastasis (17% versus 11%) [74]. Hay reported a series of 1000 patients on use of RAI and also found that RAI had no effect on recurrence or mortality in patients with MACIS score <6 [75]. After pCND, application of RAI in view of upstaged status might not provide additional benefit in this low-risk group. However, the issue of whether RAI ablation could replace pCND remains unclear and requires further studies [8]. On the other hand, because of upstaging, it is expected that more patients would require RAI ablation and inevitably they would be subjected to potential drawbacks from radiation, like salivary gland swelling, recurrent sialoadenitis, dental caries, pulmonary fibrosis, nasolacrimal outflow obstruction, and, more importantly, increased risk of second primary malignancies on a long term [76–78]. A recent single-institution study found that cumulative RAI activity = 3.0−8.9 GBq was an independent risk factor for the development of nonsynchronous second primary malignancy after adjusting for tumor stage and period of DTC diagnosis (relative risk = 2.38, *P* = 0.040) [78].

In terms of followup and long-term surveillance, since pCND results in a higher rate of postsurgical-stimulated athyroglobulinemia, the authors believe that pCND could facilitate followup and ultrasound surveillance of the neck compartments [5, 6, 8, 26]. This is particularly so if a cancer cure is defined by having athyroglobulinemia [8].

## 7. Extent of pCND and its Patient Selection

While the debate over pCND in DTC rages on, it would appear that the most rational approach would be to decide whether to perform pCND based on patients' risk stratification or on novel prognostic factors such as *BRAF* mutations with an aim of minimizing the overall surgical morbidity rate.

## 8. Extent of Surgery

According to the revised ATA guideline, either unilateral or bilateral paratracheal LN would be regarded as a formal CND but either would still result in morbidity in inexperienced hands. Since surgical morbidity is directly related to the extent of surgical dissection, a limited or unilateral pCND might seem like a logical choice [8, 20, 79]. As lymphatic spread of tumor cells tends to occur in a stepwise fashion, from thyroid gland to ipsilateral central neck first, to ipsilateral lateral neck, and later to contralateral central neck, skip metastasis leaping central neck compartment is uncommon [80]. Therefore, the ipsilateral central neck compartment is the most common site for lymph node metastasis. Ipsilateral central neck involvement occurs in up to 69%, while contralateral central neck occurs in 10~20% [81–83]. Moo et al. analyzed a cohort of 116 patients with papillary thyroid cancer undergoing thyroidectomy with pCND and none of patients with tumor size ≤1 cm have contralateral lymph node metastasis [16]. Other studies have shown that bilateral central nodal metastasis is correlated with tumor multifocality, presence of metastasis in all lateral neck levels and ipsilateral involvement of central neck dissection [84, 85]. Compared with unilateral (ipsilateral) pCND, bilateral pCND was associated with higher rate of temporary hypoparathyroidism (20.5% versus 48.0%; *P* = 0.009), (26.8% versus 48.3%; *P* = 0.021), while no significant difference in rate of postablation athyroglobulinemia (64% versus 57.1%, *P* > 0.05) or recurrence (7.1% versus 8.6%, *P* > 0.05) [86, 87]. In the authors' opinion, if one has decided on performing a pCND, an unilateral rather than a bilateral pCND would appear to be the correct choice as it provides a balance between achieving surgical completeness and avoiding significant surgical morbidity. On the other hand, for patients with high risk factors such as multifocality or ipsilateral central or lateral lymph node metastasis, bilateral pCND would seem to be more appropriate given the higher incidence of contralateral central lymph node involvement [84, 85].

Cervical thymus is occasionally removed in the central neck dissection [29]. But thymectomy is associated with increased risk of transient hypocalcaemia [20]. This is because of extensive dissection leading to devascularization of inferior parathyroid gland or unintentional removal of inferior parathyroid located in thyrothymic ligament or thymus (41%) [88]. Similar to paratracheal dissection, bilateral cervical thymectomy results in higher rate of temporary hypocalcaemia (35.5% versus 10.7%; *P* < 0.001), while contralateral intrathymic lymph node metastasis is rare. Therefore, unilateral cervical thymectomy would seem appropriate [89].

## 9. Patient Selection

Large tumor size, extrathyroidal extension, and multifocality are associated with increased risk of central neck lymph node metastasis [90]. These tumors are more likely to harbor metastasis in central neck compartment. On the other hand, large tumor size, gross extrathyroidal extension and adverse histological features and ipsilateral central neck metastasis are also poor risk factors for recurrence [91]. On selecting patients for pCND, these factors should therefore be considered at the time of operation [91].

Sentinel lymph node dissection has been advocated in management of breast and melanoma [92, 93], but its application in DTC remains limited and underinvestigated. This is because firstly a pCND can be performed using the same neck incision as the thyroidectomy and the surgical morbidity is still relatively low when compared to other lymph node dissections (e.g., axillary or groin dissection). Nevertheless, in theory, it is believed that lymphatic metastasis occurs in stepwise manner, and, by removing the first draining lymph node(s), it could predict the regional pathological lymph node status. Some proponents suggested that it could allow for more accurate staging, better patient selection to undergo formal CND and RAI ablation, and identification of sentinel lymph node outside central compartment [93]. Some authors commented that they encountered difficulties with injecting or mapping label material into small nodule in a peritumoral fashion, particularly when the tumor involved the whole lobe. For tumors in a multinodular goiter, it is also difficult to identify the site for injection and injections could lead to rupture of cystic lesion [94]. Early small single-center studies reported a high false-negative rate for SLN detection of up to 12 ~ 22% with blue dye injection or radio-isotope injection [95, 96]. And, more importantly, performing sentinel lymph node biopsy did not seem to reduce recurrence [97].

Nevertheless, a recent meta-analysis including 24 studies on sentinel node biopsy for thyroid cancer showed promising results. They found the overall false negative rate for blue dye, radio-isotopes, and combined methods were 7.7, 16, and 0 percent, respectively. The false negative in frozen section of SLN was 12%, which is comparable to sentinel lymph node biopsy examination for breast cancer. According to the results, more than half of patients with clinically negative lymph nodes could potentially avoid prophylactic neck dissection [97].

In addition to lymph node metastasis, older age, extrathyroidal extension, aggressive histology subtype, distant metastasis, and presence of *BRAF* mutation are associated with higher recurrence [98–100]. Presence of *BRAF* mutations is associated with higher risks in nodal involvement and recurrence [99–101]. New technologies are now available to test the *BRAF* status in FNA sample. Perhaps, by detecting *BRAF* mutations preoperatively, surgeons would be able to decide which patients should undergo pCND and total thyroidectomy and which should undergo total thyroidectomy alone [102, 103]. However, further studies are required.

## 10. Difficulties and Biases with Conducting Studies on pCND in DTC

Since there is no prospective randomized trial to evaluate the efficacy of pCND, evidence on pCND comes from case series or retrospective cohort or analysis. The difficulty of conducting a prospective study could be explained by its indolent nature of DTC, as most patients live for more than 10 years [104] and recurrence may occur up to 20 years after primary diagnosis [67, 105]. Perhaps, future studies could evaluate the potential role of molecular markers such as VEGF-C to predict lymph node recurrences [106]. Therefore, evaluating survivals as endpoints might be difficult and unrealistic. The natural history of DTC would be for some patients to undergo multiple neck operations and that results in a negative impact on quality of life. Therefore, assessing the number of recurrences and surgical morbidity within a cohort might be more realistic in patients with DTC. However, a large number of cases following up over a long period of time would be needed to detect a small difference (i.e., power of the study). The second problem relates to the indication for pCND as there is often individual surgeon's bias. Though recent studies have designated nature of pCND as therapeutic or prophylactic in nature, the decision for pCND was mostly at the discretion of the operating surgeon [6, 8, 65]. Patients who underwent pCND were generally more likely to have more risk factors such as larger tumor size, extrathyroidal extension, and multifocality which might have been easily detectable during operation [8, 65]. High-risk patients are generally more likely to undergo pCND, and this may attenuate the potential benefit of pCND. Furthermore, disease status is upstaged in up to 30% of patients undergoing pCND, and this in turn increases the overall proportion of patients receiving RAI ablation [6, 8].

## 11. Conclusion

There is little good evidence to support the routine practice of pCND in patients with DTC undergoing a total thyroidectomy. Although the recent revised ATA guideline has clarified many inconsistencies and has recommended the practice of pCND based on tumor risks and stages, many issues and controversies surrounding the subject of pCND in DTC remain. Nevertheless, in the recent literature, there has been a trend toward lower recurrence rate in those with pCND when compared to those without pCND. However, this is subjected to biases and there is always the concern of its applicability in the wider surgical community, as the outcomes of pCND are volume-related. An ipsilateral unilateral pCND would appear to provide a good balance between achieving surgical completeness and avoiding adding significant surgical morbidity if pCND was indicated. Balancing the surgical morbidity and long-term benefits as well as better patient selection (i.e., high tumor-risk groups) to undergo pCND would be the keys. Further studies on this controversial subject are much needed.

## Figures and Tables

**Table 1:** A summary of the arguments for and against prophylactic neck dissection (pCND) during total thyroidectomy for differentiated thyroid carcinoma.

Arguments for pCND	Arguments against pCND
Subclinical central lymph node metastasis is common	Only a small proportion of subclinical central lymph node metastasis would develop clinically significant recurrences
Lymph node metastasis leads to higher recurrences and poorer survival	There is no level-one evidence to suggest that pCND could improve survival
pCND may reduce recurrence and lower postoperative thyroglobulin levels	Tumor upstaging leads to more radio-iodine ablation which might not be necessary
Preoperative and intraoperative evaluations of central compartment lymph node metastasis are not reliable	Operation in recurrent case could be safely performed by experience hands
Improved tumor staging and stratification of tumors	Majority of thyroidectomy are performed by low-volume surgeons
Reduce the need for reoperation in central neck recurrence which is associated with greater morbidity	Increased surgical morbidities (hypoparathyroidism and recurrent laryngeal nerve injury)
pCND can be safely performed with comparable morbidity to thyroidectomy alone in experience hands	

**Table 2:** A comparison of cancer-specific mortality between those who underwent a total thyroidectomy and prophylactic neck dissection (CND+ group) and those who underwent a total thyroidectomy only (CND− group).

First author/year	Number of patients	Followup duration (months)	Cancer-specific mortality	*P *value	First author/year	Number of patients
	CND+ group	CND− group		CND+ group	CND− group	
Tisell/1996 [28]	195	199 [62]; 167 [63]	156 months (median)	1.6%	8.4%–11.1%	Not reported
Sywak/2006 [5]	P-56, A	391	CND−: 70 months CND+: 25 months (median)	0%	0%	Not significant
Roh/2007 [23]	P-40, B T-42 (26/42 with lateral neck dissection)	73	52 months (mean)	0%	0%	Not significant

CND−: thyroidectomy alone, CND+: thyroidectomy plus central neck dissection; P: prophylactic, T: therapeutic; A: unilateral, B: bilateral.

**Table 3:** A comparison of recurrence rates between those who underwent a total thyroidectomy and prophylactic neck dissection (CND+ group) and those who underwent a total thyroidectomy only (CND− group).

Study design	First author/year	Number of patients		Followup (mean)	Overall recurrence		*P* value
		CND+ group	CND− group		CND+ group	CND**−** group	
Retrospective	Gemsenjäger/2003 [58]	P-29	88	8.1 years	3.4%	2.3%	Not mentioned
Retrospective	Wada/2003 [57]	P-235	155	53 months	0.4%	0.6%	Not significant
Retrospective	Sywak/2006 [5]	P-56, A	391	CND−: 70 months CND+: 24.5 months (median)	3.6%	5.6%	Not mentioned
Retrospective	Roh/2007 [23]	P-40, B T-42, B	73	52 months	P-0% T-1.2%	4.0%	Not mentioned
Retrospective	Zungia/2009 [65]	P-136,B	130	6.9 years	5 yr DFS-88.2%	5 yr DFS-85.6%	0.72
Retrospective	Costa/2009 [67]	P-126, B	118	CND−: 64 months CND+: 47 months	6.3%	7.7%	0.83
Retrospective	Moo/2010 [24]	P-45, B	36	3.1 years	4.4%	16.7%	0.13
Meta-analysis	Zetoune/2010 [66]	P-396, A/B	868		2.0%	3.9%	NS
Retrospective	Lang/2011 [8]	P-82, A	103	26 months (median)	3.7%	2.9%	1.0

P: prophylactic, T: therapeutic; A: unilateral, B: bilateral; 5 yr DFS: 5-year disease-free survival.

**Table 4:** A comparison of surgical-related morbidities between those who underwent a total thyroidectomy and prophylactic neck dissection (CND+ group) and those who underwent a total thyroidectomy only (CND− group).

First author/year	Number of Patients		Transient hypoparathyroidism (%)		Permanent hypoparathyroidism (%)		Transient recurrent nerve injury (%)		Permanent recurrent nerve injury (%)	
	CND+	CND−	CND+	CND−	CND+	CND−	CND+	CND−	CND+	CND−
Henry/1998 [69]	P-50,B	50	14^#^	8	4.0^#^	0	4^#^	6	0	0
Steinmullar/1999 [70]	P-53, B	70	39.6	35.7	0.7	0	4^#^	6	0	0
Gemsenjäger/2003 [58]	P-29, A/B T-42, B	159			P-0 T-1.4	0	3.7	8.6	1.9	0
Sywak/2006 [5]	P-56, A	391	18*	8	1.8	0.5			P-0 T-4.2	0
Roh/2007 [23]	P-40, B T-42	73	30.5*	9.6	4.9	0	1.8	1.0	0	1
Palestini/2008 [22]	P-93, A	148	26.9*	12.8	0	2.7	7.3	4.1	3.6	2.7
Rosenbaum/2009 [27]	P-4, B T-18, B	88	86*	58	5	0	5.4*	1.4	0	1.4
Sadowski/2009 [25]	P-169, B T-11, A	130					9	2	0	1
Hughes/2010 [6]	P-78, B	65	27*	8	2.6	0	11	6	4	0
Moo/2010 [24]	P-45, B	36	31*	5	0	5^#^	0	3.1		
Shindo/2010 [21]	P-108, A T-14, B	134	13.1	25.4*	0.8	0.7	4^#^	0	0	0
Lang/2011 [8]	P-82, A	103	18.3*	8.7	2.4	1.0	0	1	0	0

*:* P* < 0.05, ^#^:* P* value not reported.

Abbreviations: P: prophylactic, T: therapeutic; A: unilateral, B: bilateral.
